# Influence of Yo-Yo IR2 Scores on Internal and External Workloads and Fatigue Responses of Tag Football Players during Tournament Competition

**DOI:** 10.1371/journal.pone.0140547

**Published:** 2015-10-14

**Authors:** Luke W. Hogarth, Brendan J. Burkett, Mark R. McKean

**Affiliations:** School of Health and Sport Sciences, Faculty of Science, Health, Education and Engineering, University of the Sunshine Coast, Maroochydore, Queensland, Australia; University of Alabama at Birmingham, UNITED STATES

## Abstract

The purpose of this study was to: a) identify changes in jump height and perceived well-being as indirect markers of fatigue, b) determine the internal and external workloads performed by players, and c) examine the influence of Yo-Yo IR2 on changes in jump height, perceived well-being and internal and external workloads during a tag football tournament. Microtechnology devices combined with heart rate (HR) chest straps provided external and internal measures of match work-rate and workload for twelve male tag football players during the 2014 Australian National Championships. Jump height and perceived well-being were assessed prior to and during the tournament as indirect measures of fatigue. Changes in work-rate, workload and fatigue measures between high- and low-fitness groups were examined based on players’ Yo-Yo IR2 score using a median split technique. The low- and high-fitness groups reported similar mean HR, Playerload^TM^/min, and distance/min for matches, however the low-fitness group reported higher perceived match-intensities (ES = 0.90–1.35) for several matches. Further, the high-fitness group reported higher measures of tournament workload, including distance (ES = 0.71), Playerload^TM^ (ES = 0.85) and Edwards’ training impulse (TRIMP) (ES = 1.23) than the low-fitness group. High- and low-fitness groups both showed large decreases (ES = 1.46–1.49) in perceived well-being during the tournament, although jump height did not decrease below pre-tournament values. Increased Yo-Yo IR2 appears to offer a protective effect against player fatigue despite increased workloads during a tag football tournament. It is vital that training programs adequately prepare tag football players for tournament competition to maximise performance and minimise player fatigue.

## Introduction

Tag football is an international game that is best described as a modified version of rugby league, where players aim to remove Velcro tags attached to the each players’ shorts rather than tackling their opponent. A typical match is contested by two teams of sixteen players (with 8 players on the field at any one time) over two 20 min playing halves on a 70 m by 50 m playing field. Teams have unlimited interchanges during a match with the frequency and duration of interchanges often being self-regulated by the playing group. Elite competition is played in tournament format at state-, national- and international-representative levels where players may be required to compete in up to four matches on a single day, over two to three consecutive days of competition. The intensified nature of these tournaments likely place considerable demands on players that may affect their physical performance during the latter stages of competition.

The identification of players’ neuromuscular function and perceived levels of fatigue may provide important insights into their fatigue-recovery cycle during tournament tag football [1, 2]. Several studies have demonstrated the usefulness of cost-effective questionnaires that rate players’ perceived levels of fatigue and well-being following competitive match-play and during intensified competition [3–5]. Further, previous research in tag football has shown players experience large decreases in perceived well-being during consecutive tag football matches played on the same day, and demonstrated moderate negative correlations between decreased perceived well-being and tournament high-speed running distance (r = -0.32) and effort frequency (r = -0.44) [6]. Questionnaires that rate players’ perceptual fatigue and well-being may provide important information on changes in their fatigue-state during tournament tag football.

Measures of neuromuscular function have typically been assessed using jump tests, as they incorporate the stretch-shortening cycle, are easy to administer in team settings and are unlikely to induce further increases in fatigue [7]. Studies have shown force-time derived measures of jump performance, such as flight time-to-contraction time (FT: CT), to provide sensitive measures of neuromuscular fatigue following team sport match-play [8–10] and tournament competition [5, 11]. Jump height provides another measure of jump performance that may be more practically convenient as it can be assessed without force platform equipment, although studies have questioned the sensitivity of jump height to assess neuromuscular fatigue following team sport match-play [8, 12]. However, other studies examining neuromuscular fatigue responses during tournament competition have reported players to experience decreased jump height. These studies have also shown the amount of jump height decrease is associated with external match workloads, such as playing time, high-speed running distance and maximal acceleration and deceleration frequency [6, 13, 14]. Therefore, changes in jump height may be associated with the internal and external workloads performed by players during tag football matches, and may provide a sensitive measure of neuromuscular fatigue during tournament competition.

Advancements in player tracking technology have allowed for the internal and external match-activity profiles of team sport athletes to be readily assessed, providing coaches with important information used to prescribe training. Measures of workload are classified as external or internal; with external load referring to the work completed by an athlete and internal load being the relative physiological or psychological stress imposed [1]. The use of microtechnology devices incorporating global positioning system (GPS) and accelerometer technology have become common practice in team sports to identify the external workloads placed on players during training and competition, as well as the workload performed relative to the duration of training or match-play (i.e. work-rate) [15]. Traditionally, the distance covered during training or competition has been used as a key measure of external workload, however, this measure may be insensitive to changes in running velocity, jumping and physical impacts that place considerable stress on the body. More recently, the incorporation of GPS with high resolution tri-axial accelerometers has provided another metric known as Playerload^TM^, which is an arbitrary unit derived from 3-dimensional measures of the instantaneous changes of acceleration [16]. Boyd et al. [17] have demonstrated Playerload^TM^ to be a reliable measure using a mechanical shaker, reporting within- and between-unit coefficients of variation (CV) of 0.91–1.05% and 1.02–1.10%, respectively. The inclusion of Playerload^TM^ to examine the external workloads of tag football players during tournament competition may provide a more sensitive measure associated with fatigue-related changes compared to GPS-derived measures of distance.

In addition to external workload, an understanding of the physiological and psychological stress, or internal workload, is necessary to understand the demands placed on athletes during competition. Internal workload during team sport competition has been assessed using questionnaires that rate players perceived effort or match-intensity (RPE) [18, 19], heart rate (HR) monitoring and training impulse (TRIMP) measures [18, 19], and increases in biochemical and hormonal markers such as blood lactate [10, 20]. Identifying the internal workload is necessary to understand how the external workload performed by an athlete impacts their fatigue-recovery cycle. For instance, an athlete may maintain match work-rate (e.g. m/min) between consecutive matches, however, depending on the fatigue-state of the athlete, this may be achieved through higher perceived effort [5]. Further, an examination of the relationship between physiological and perceptual indicators of internal load (e.g. HR-RPE ratio) reported at a given exercise intensity may also provide information on the fatigue-state of an athlete [1, 21]. A combination of external and internal workload measures are likely to provide the most detailed insight into the demands of tournament competition and the associated fatigue responses experienced by players.

There is limited information on the physical and physiological demands of tournament tag football and its impact on players’ fatigue-recovery cycle. Although previous research has shown increased measures of neuromuscular and perceptual fatigue to be associated with reduced external work-rates during consecutive tag football matches [6], the internal workload responses of players has yet to be determined and it is unclear how external and internal workload measures interact with player’s fatigue response. Further, it is unknown whether physical capacity influences the internal and external workloads performed by players and their fatigue responses during tournament tag football. Previous research in rugby sevens has found state-level players experience higher levels of fatigue during tournament competition despite lower external workloads [11]. This disparity may be attributed to differences in physical fitness, as demonstrated in rugby league where players with high-fitness experience less fatigue following single matches [22] as well as intensified tournament competition [23]. Previous research has shown Yo-Yo intermittent recovery test level 2 (Yo-Yo IR2) to be associated with external measures of work-rate during tag football matches suggesting that it may be a useful test to assess player readiness and fitness associated with tag football match-play [24]. Detailing the relationship between the physical capacities assessed by the Yo-Yo IR2 and player workloads and fatigue responses during tournament competition may have important implications for athlete testing and training practices in tag football.

The purpose of this study was to: a) identify changes in jump height and perceived well-being as indirect markers of fatigue, b) determine the internal and external workloads performed by players, and c) examine the influence of Yo-Yo IR2 on changes in jump height, perceived well-being and internal and external workloads during a tag football tournament.

## Materials and Methods

### Study Design

A prospective and observational study design was employed. Activity profiles and physiological responses were determined using microtechnology devices incorporating global positioning system (GPS) and accelerometer technology combined with polar heart rate (HR) chest straps for five matches played over two days. Jump height and perceived well-being were examined as indirect measures of fatigue prior to and during the tournament using a vertical jump test and subjective questionnaire, respectively. Differences in perceived well-being, jump height, and internal and external measures of workload and work-rate were examined based on players’ Yo-Yo IR2 performance.

### Participants

Twelve male tag football players participated in this study (age 24±4 yrs; body mass 74.9±10.3 kg; height 1.78±0.05 m; estimated VO_2max_ 59.7±5.2 ml.kg^-1^.min^-1^). Participants were from the same team that placed second out of sixteen teams that competed in the open men’s division of the 2014 Australian National Championships. The Australian National Championships is a three day tournament contested by regional-representative teams from Australia and New Zealand. It is the highest standard of tag football competition played in Australia and provides players with a pathway to represent Australia during the Triennial Tag Football World Cup. All players and coaching staff of the participating team provided their written informed consent. Procedures were approved by the University of the Sunshine Coast’s Human Research Ethics Committee (HREC) in the spirit of the Declaration of Helsinki, HREC approval number: S/13/471.

### Procedures

Yo-Yo IR2 was assessed 5 days prior to the tournament before the team’s final combined squad training following a 2-day unloading phase. On arrival, participants completed the perceptual well-being questionnaire and vertical jump test which they had completed regularly during previous training sessions. Players then completed the Yo-Yo IR2 before commencing usual squad training activities. The testing procedures for the Yo-Yo IR2 have been described in detail elsewhere [25]. All players performed the test at the same time of day on an outdoor playing surface. Players were familiar with the Yo-Yo IR2 which they had performed several times during previous training sessions. Players were categorised into low- (n = 6; Yo-Yo IR2 = 747±60 m) and high-fitness groups (n = 6; Yo-Yo IR2 = 1373±278 m) based on their Yo-Yo IR2 score using a median-split technique. The Yo-Yo IR2 was shown to have acceptable reliability within this study’s participant cohort (CV = 6.6%; ICC = 0.96).

**Fig 1:**
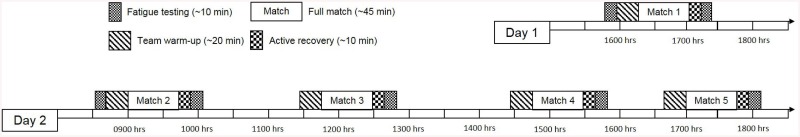
Fatigue monitoring and match schedule for the participating tag football team during the tournament.

The time-course of perceptual well-being and jump height assessment during the tournament is presented in Fig 1 along with the team’s match schedule. Perceived well-being and jump height was assessed approximately 30 min prior to the first match on days one and two, and 10–15 min following each match. Perceived well-being was assessed using a previously recommended questionnaire that sums players’ ratings of fatigue, sleep quality, general muscle soreness, stress and mood on a 5-point Likert scale, with higher scores indicating more optimal feelings of perceived well-being [5, 26]. Jump height was assessed during three maximal vertical jump attempts using a Swift Yardstick apparatus (Swift Performance Equipment, Australia) following a standardised warm-up involving dynamic stretching and jumping activities. Players were instructed to perform a rapid countermovement jump from a standing start. Movement of arms was not restricted during the jump attempts and the depth of the countermovement action was self-selected. Jump height assessed using the vertical jump test was shown to have acceptable reliability in this study’s participant cohort (CV = 3.5%; ICC = 0.91).

Following the assessment of perceived well-being and jump height players were fitted with microtechnology devices (MinimaxX S4, Catapult sports, Australia) incorporating GPS sampling at 10 Hz and accelerometer technology sampling at 100 Hz. Each player wore the same microtechnology device for the duration of the tournament and were also fitted with HR chest straps (Heart Rate Sensor H1, Polar, Finland). Players were familiar with both the microtechnology device and HR chest strap which they had worn previously during training and competition on numerous occasions prior to the tournament. Players performed a standardised warm-up before being ready to take the field approximately 2 min prior to scheduled kick-off.

Data was compiled and analysed in Sprint 5.1 (Catapult Sports, Australia). Periods of time where players were off the field, including interchanges and half-time periods, where excluded from analysis. Match-activity profile variables, including distance and Playerload^TM^, were expressed as match workloads (distance and Playerload^TM^) and work-rates relative to players’ on-field time (distance/min and Playerload^TM^/min). Distance was further categorised into low-speed running (LSR = 0.4–14.0 km/h) and high-speed running (HSR = ≥14.1 km/h) as previously recommended for tag football [27, 28]. Playerload^TM^ describes the vector magnitude formulated from accumulated accelerometer data in all three axes (anterioposterior, mediolateral, and craniocaudical) and is calculated by the manufacturer’s software [16, 17]. In addition to work-rate and workload measures, the number of field rotations performed by each player was recorded. A single field rotation was defined as a time period spent on-field during match-play prior to following an interchange rest period.

Mean match HR was expressed as a percentage of players peak HR (%HR_peak_), determined as players’ peak HR reached during the Yo-Yo IR2. Edwards’ training impulse (TRIMP) was calculated from HR recordings as a measure of internal match load. Edwards’ TRIMP was calculated as: duration in zone 1·1 + duration in zone 2·2 + duration in zone 3·3 + duration in zone 4·4 + duration in zone 5·5; where Zone 1 = 50% to 60% HR_peak_, Zone 2 = 60% to 70% HR_peak_, Zone 3 = 70% to 80% HR_peak_, Zone 4 = 80% to 90% HR_peak_, and Zone 5 = 90% to 100% HR_peak_ [19]. Additionally, perceptual match-intensity was quantified using a modified Borg’s rate of perceived exertion (RPE) scale (CR-10). Players’ RPE score was collected 10–15 minutes following each match prior to perceived well-being and jump height assessment [29]. Players were asked to point to the RPE scale so that their score was blinded from other participants to avoid bias. The RPE score was then multiplied by player’s on-field time as an additional measure of internal workload (Session-RPE) [30]. Further, mean match HR to RPE ratio (HR-RPE ratio) was calculated to indicate changes in the relationship between physiological and perceptual load across matches [21]. Players were familiarised with the Borg’s RPE scale to assess perceptual match-intensity prior to the commencement of the tournament.

### Statistical Analyses

Data were checked for normality of distribution using a Shapiro-Wilk test. Non-normally distributed data were log transformed prior to statistical analysis to reduce the non-uniformity of error and back transformed to attain descriptive statistics (mean±SD). Standardised differences were calculated to determine differences in jump height, perceived well-being, and workload variables between matches and groups (high- and low-fitness). The precision of estimates is indicated with 90% confidence limit (CL). The magnitude of the difference was assessed as trivial (<0.2), small (0.21–0.6), moderate (0.61–1.2), large (1.21–2.0) and very large (>2.1) as per previously standardised criteria [31]. The effect was reported as unclear when the CL crossed the threshold for both substantially positive (0.2) and negative (-0.2) values [31]. Pearson’s correlation coefficients were calculated to examine the relationship between Yo-Yo IR2, jump height, perceived well-being, and match work-rates and workloads. Pearson’s correlation coefficients were reported as small (0.1–0.3), moderate (0.3–0.5), large (0.5–0.7), very large (0.7–0.9) and almost perfect (0.9–1.0) [32].

## Results

### Yo-Yo IR2 scores, match opposition and outcome

The mean Yo-Yo IR2 score for the combined playing group was 1060±379 m. The high- and low-fitness groups had a Yo-Yo IR2 score of 1373±278 m and 747±60 m, respectively. The opposition ranking, score and the mean RPE score for matches are presented in Table 1. There was a very large negative correlation between opposition ranking and match RPE (r = -0.88, p<0.01).

**Table 1:** Opposition ranking and score for matches during the tournament.

		Opposition rank	Score	RPE
**Day 1**	**Match 1**	2	2–4	5.5 (3–7)
**Day 2**	**Match 2**	6	10–1	3.6 (3–4)
	**Match 3**	5	5–0	5.0 (4–6)
	**Match 4**	1	3–4	5.8 (5–8)
	**Match 5**	4	8–3	4.3 (3–5)

Opposition rank is based on team ranking following round matches. RPE data; mean (range).

### Internal and external work-rates

**Fig 2:**
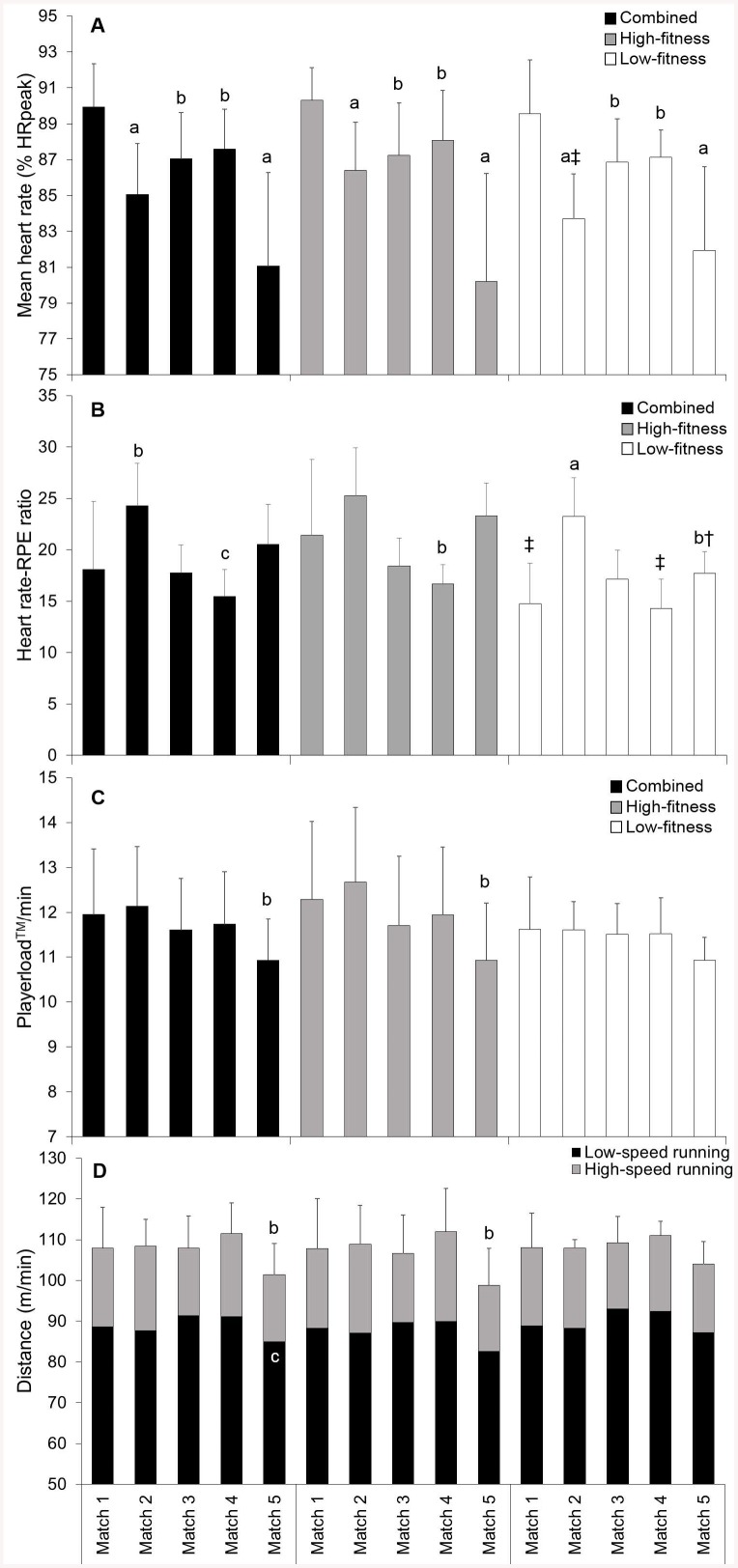
Changes in mean HR (A), HR-RPE ratio (B), Playerload^TM^/min (C) and distance/min (D) during the tournament for players grouped into high- and low-fitness groups. *a*, *b* and *c* indicate a large, moderate and small difference compared to match 1, respectively. † and ‡ indicate a large and moderate difference compared to the high-fitness group, respectively.

There were small to moderate decreases in distance/min (ES = 0.70), LSR distance (ES = 0.53) and Playerload^TM^/min (ES = 0.78) from the first to final match (Fig 2). There were also moderate to large reductions in mean _HR_ following the first match (ES = 0.92–1.47), with the lowest values reported during the final match (ES = 1.47). There were no meaningful differences between high- and low-fitness groups except for mean HR values during match 2 (ES = 0.95), although the low-fitness group reported lower HR-RPE ratios for matches 1, 4 and 5 (Fig 2B). The low-fitness group maintained distance/min and Playerload^TM^/min over the five matches, whilst the high-fitness groups showed moderate reductions in distance/min (ES = 0.80) and Playerload^TM^/min (ES = 0.84) during the final match. There were small to large correlations (r = 0.21 to 0.63) showing Yo-Yo IR2 to be positively associated with match work-rate measures.

### Internal and external workloads

The high-fitness group reported higher Playerload^TM^ for the tournament than the low-fitness group (ES = 0.85), and also covered greater total distance (ES = 1.45) and Playerload^TM^ (ES = 1.47) during match 4 (Table 2). There were also moderate to large differences in field time (ES = 1.59) and field rotation times (ES = 1.11) between the low- and high-fitness groups for match 4. Further, Yo-Yo IR2 score was positively associated with tournament distance (r = 0.43, moderate), Playerload^TM^ (r = 0.63, large) and Edwards’ TRIMP (r = 0.33, moderate) and negatively associated with session-RPE (r = -0.59, large). Players showed moderate reductions in total distance (ES = 0.71), Playerload^TM^ (ES = 0.77), Edwards’ TRIMP (ES = 1.09) and session-RPE (ES = 0.88) during the final match.

**Table 2:** Tournament and match workloads for tag football players grouped into high- and low-fitness groups.

		Tournament	Match 1	Match 2	Match 3	Match 4	Match 5
**Field time (min)**							
	**Combined**	103.2 ± 5.9	20.5 ± 2.3	20.6 ± 1.5	20.8 ± 3.9	20.7 ± 2.0	20.7 ± 1.9
	**High-fitness group**	105.7 ± 6.1	20.2 ± 2.5	20.9 ± 1.8	21.6 ± 3.6	22.3 ± 1.4*b*	20.8 ± 1.9
	**Low-fitness group**	100.7 ± 4.9‡	20.8 ± 2.4	20.4 ± 1.4	19.9 ± 4.4	19.1 ± 1.0†*b*	20.5 ± 1.9
**Field rotation time (min)**							
	**Combined**	3.7 ± 0.4	3.6 ± 0.7	3.3 ± 0.4	4.0 ± 0.8	3.9 ± 0.6	3.6 ± 0.5
	**High-fitness group**	3.8 ± 0.2	3.6 ± 0.7	3.4 ± 0.3	4.1 ± 0.7	4.2 ± 0.6*b*	3.8 ± 0.5
	**Low-fitness group**	3.5 ± 0.4	3.6 ± 0.7	3.3 ± 0.5	3.9 ± 1.0	3.5 ± 0.5‡	3.4 ± 0.5‡
**Distance (m)**							
	**Combined**	11047 ± 553	2195 ± 165	2233 ± 170	2229 ± 387	2305 ± 251	2086 ± 125*b*
	**High-fitness group**	11243 ± 494	2153 ± 146	2269 ± 196	2292 ± 375	2487 ± 192*a*	2042 ± 110*b*
	**Low-fitness group**	10851 ± 579	2238 ± 186	2196 ± 150	2166 ± 424	2122 ± 148†	2129 ± 133
**Playerload** ^**TM**^ **(AU)**							
	**Combined**	1201 ± 120	243 ± 26	250 ± 33	240 ± 48	242 ± 31	225 ± 18*b*
	**High-fitness group**	1252 ± 127	245 ± 27	265 ± 38	251 ± 48	265 ± 24	226 ± 18*b*
	**Low-fitness group**	1150 ± 96‡	241 ± 26	236 ± 20‡	229 ± 48	220 ± 17†*b*	224 ± 20
**Edwards’ TRIMP (AU)**							
	**Combined**	430 ± 28	91 ± 11	84 ± 10*b*	89 ± 16	89 ± 10	76 ± 12*b*
	**High-fitness group**	447 ± 24	93 ± 13	89 ± 8	93 ± 15	98 ± 5	74 ± 10*a*
	**Low-fitness group**	413 ± 21†	89 ± 10	80 ± 10‡*b*	85 ± 18	81 ± 5†*b*	78 ± 15*b*
**Session RPE (AU)**							
	**Combined**	494 ± 64	113 ± 37	74 ± 13*b*	103 ± 22	120 ± 22	84 ± 18*b*
	**High-fitness group**	463 ± 55	94 ± 34	74 ± 16	105 ± 27	119 ± 17*b*	72 ± 10*b*
	**Low-fitness group**	526 ± 61‡	132 ± 32‡	74 ± 10*a*	102 ± 19*b*	121 ± 28	96 ± 17†*b*

Data are mean ± standard deviation. TRIMP = training impulse. RPE = rating of perceived exertion.

† and ‡ indicate a large (ES >1.2) and moderate (ES = 0.6–1.2) difference compared to the high-fitness group, respectively.

*a* and *b* indicate a large (ES >1.2) and moderate (ES = 0.6–1.2) difference compared to match 1, respectively.

### Perceived well-being

**Fig 3:**
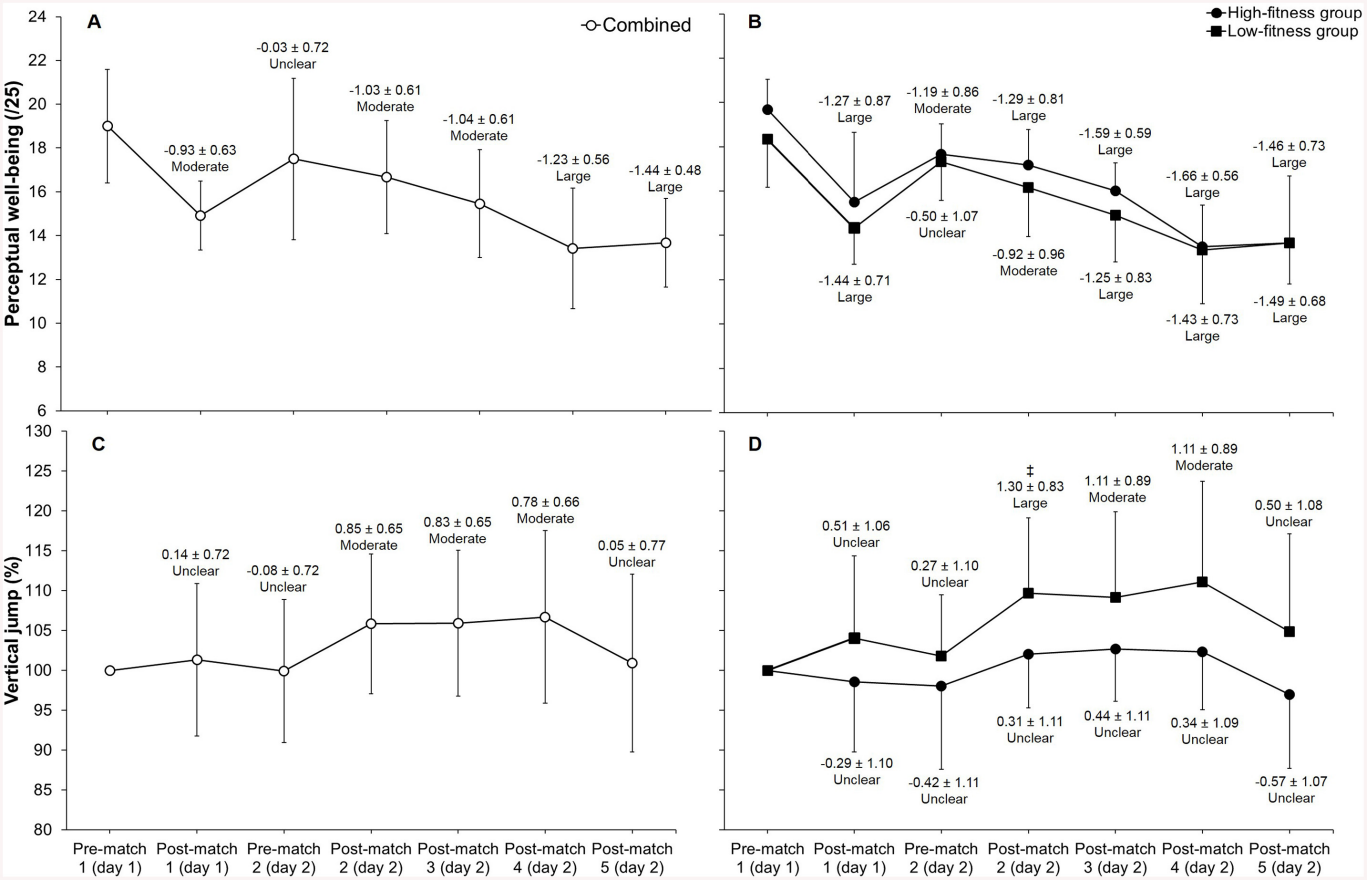
Changes in perceived well-being (A and B) and jump height (C and D) for tag football players grouped into high- and low-fitness groups during the tournament. ‡ indicates a moderate difference between low- and high-fitness groups.

**Fig 4:**
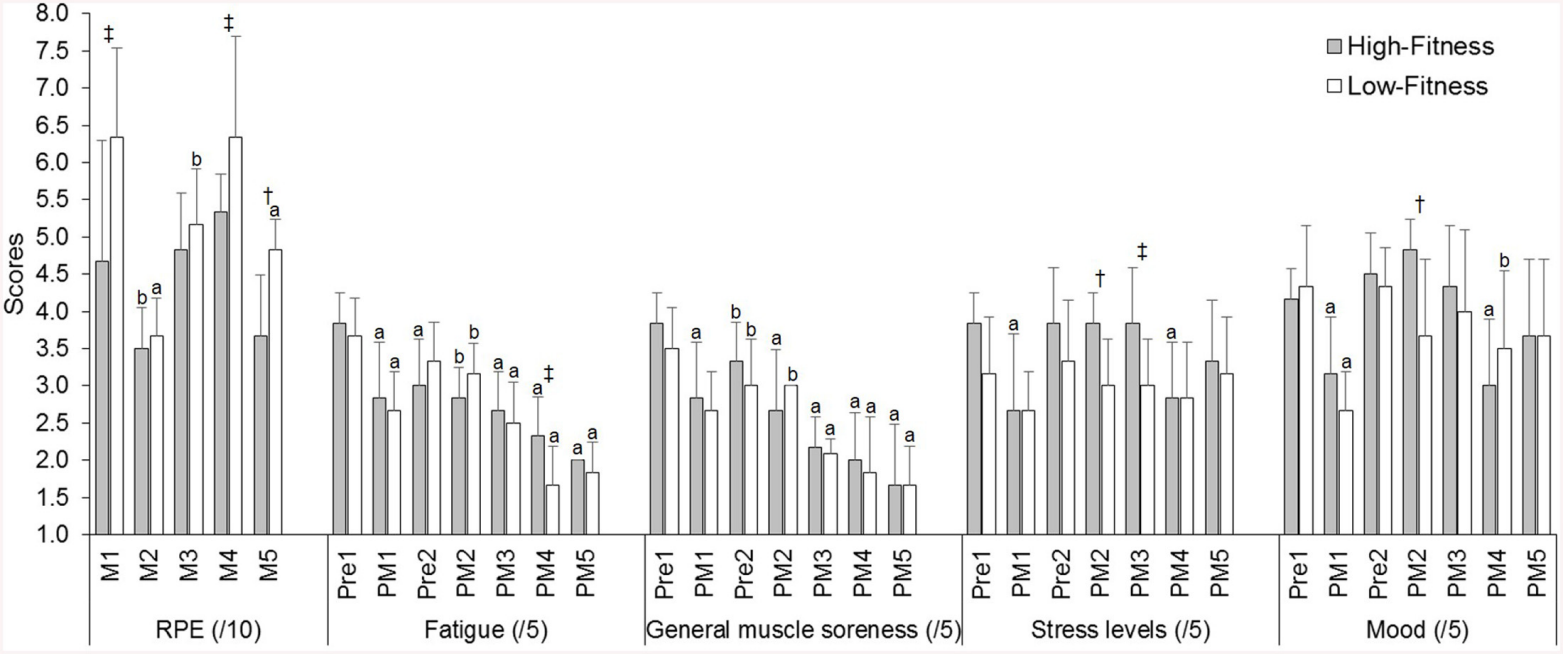
Changes in perceived match-intensity (RPE) and feelings of fatigue, general muscle soreness, stress levels and mood during the tournament. *a* and *b* indicate a large and moderate difference compared to match 1, respectively. † and ‡ indicate a large and moderate difference between low- and high-fitness groups, respectively.

Players reported moderate to large reductions in perceived well-being following matches compared to pre-tournament values (ES = 0.93–1.44), with the lowest values reported following matches 4 and 5 (Fig 3A). High- and low-fitness groups showed similar changes in perceived well-being during the tournament (Fig 3B), although there were a number of moderate and large differences between groups for perceived fatigue, stress levels and mood following matches (Fig 4). Changes in perceived well-being (pre-match 1 vs. post-match 5) were correlated with changes in distance/min (r = 0.52, large), Playerload^TM^/min (r = 0.46, moderate) and mean HR (r = 0.50, large) from the first to final match. The low-fitness group also reported higher perceived match-intensities (RPE) for matches 1, 4 and 5 compared to the high-fitness group (Fig 4).

### Jump height

Players’ jump height was 57.3±5.6 cm at pre-match 1, 57.9±7.0 cm post-match 1, 57.0±5.5 cm pre-match 2, 60.4±5.9 cm post-match 2, 60.4±5.5 cm post-match 3, 60.9±6.9 cm post-match 4, and 57.5±6.1 cm post-match 5. Jump height was expressed relative to each player’s pre-match 1 score to account for individual variances (Fig 3C and 3D). Players showed moderate increases in jump height following matches 2, 3 and 4 compared to pre-tournament values (Fig 3C). There were small reductions in jump height post-match 5 compared to post-match 2, 3 and 4 values (ES = 0.49–0.52), although jump height did not deviate substantially from pre-match 1 values following the final match. The low- and high-fitness groups reported similar jump height during the tournament, except for match 2 (Fig 3D).

## Discussion

This study aimed to: a) identify changes in jump height and perceived well-being as indirect markers of fatigue, b) determine the internal and external workloads performed by players, and c) examine the influence of Yo-Yo IR2 on changes in jump height, perceived well-being and workload and work-rate measures during a tag football tournament. There were a number of interesting findings of this study that may have important implications for coaching staff working with tag football players. First, high- and low-fitness groups reported similar match work-rates during the tournament, although the low-fitness group reported higher perceived match-intensities (RPE) and lower HR-RPE ratios for several matches. Second, Yo-Yo IR2 was positively correlated with external (distance and Playerload^TM^) and physiological (Edwards’ TRIMP) measures of tournament workload, whilst being negatively correlated with psychological measures of tournament workload (Session-RPE). Third, players reported large decreases in perceived well-being during the tournament, which did not appear to be associated with Yo-Yo IR2 and was mostly attributed to progressive increases in feelings of fatigue and general muscle soreness. Finally, jump height initially increased following matches on the second day, and did not decrease compared to pre-tournament values during the tournament.

Players showed reduced internal and external measures of match work-rate during the final match (Fig 2), perhaps as a result of increased perceptual fatigue and general muscle soreness on the second day of competition (Fig 4). Both low- and high-fitness groups recorded peak mean HR during match 1 and experienced moderate to large decreases for matches 2 to 5 (Fig 2A). Whilst low-fitness players maintained external work-rate measures (distance/min and PlayerloadTM/min) over the five matches, high-fitness players reported small to moderate decreases in distance/min, LSR m/min and Playerload^TM^/min during the final match (Fig 2C and 2D). These results may suggest that accumulated fatigue caused the high-fitness group to down-regulate their match work-rates to avoid further increases in fatigue [6, 33]. However, there were no clear differences in match work-rate or perceived well-being scores following the final match between low- and high-fitness groups, and the high-fitness group reported lower perceived match-intensity (RPE) during the final match compared to previous matches on the same day (Fig 4). Therefore, the decreased match work-rate performed by the high-fitness group during the final match is more likely explained by factors such as match opposition and score line rather than increased neuromuscular or perceptual fatigue [34, 35]. Indeed, there was a very large negative correlation found between opposition ranking and mean RPE (r = -0.88, p<0.01) (Table 1). Further, despite reporting similar match work-rates and perceived well-being scores post-match, the low-fitness group reported higher perceived match-intensity (RPE; ES = 1.35) and a lower HR-RPE ratio (ES = 1.43) for match 5 than the high-fitness group (Fig 2B and Fig 4). These findings suggest that increased Yo-Yo IR2 may allow players to maintain external measures of match work-rate whilst reducing their perceived match-intensity during tag football matches played in tournament format.

This study found a number of differences between low- and high-fitness groups for internal and external workloads performed during matches (Table 2). The high-fitness group reported longer field times and performed higher Playerload^TM^ and Edwards’ TRIMP for the tournament compared to the low-fitness group that reported higher session-RPE (Table 2). Similarly, Yo-Yo IR2 was positively correlated with tournament distance (r = 0.43, moderate), Playerload^TM^ (r = 0.63, large) and Edwards’ TRIMP (r = 0.33, moderate) whilst being negatively correlated with session-RPE (r = -0.58, large). These results indicate that Yo-Yo IR2 contributes to higher external and physiological measures of workload during tournament tag football whilst minimising the amount of psychological stress reported by players. It is also interesting to note that the high-fitness group reported higher physiological (Edwards’ TRIMP) and external (distance and Playerload^TM^) measures of workload for matches 2 and 4 (Table 2), despite the high- and low-fitness groups performing similar match work-rates (Fig 2). As interchanges are self-regulated by the playing group in tag football, these results can be explained by players in the low-fitness group moderating their interchange strategy during the more intense matches (e.g. match 4; Fig 2 and Fig 4), perhaps to avoid transient fatigue and maintain on-field work-rates. Indeed, the high-fitness group reported longer field rotation times during the final two matches of the tournament (Table 2), where the low-fitness group reported higher percieved match-intensities and players’ ratings of fatigue and general muscle soreness were most evident (Fig 2B and Fig 4). Therefore, the Yo-Yo IR2 appears to be associated with a player’s capability to maintain external measures of match work-rate during longer field rotations and perform higher external workloads during the more intense matches played towards the end of a tag football tournament.

Players reported large decreases in perceived well-being during the tournament which did not appear to be influenced by Yo-Yo IR2 (Fig 3A and 3B). The largest decrease in perceived well-being was reported following match 1 on the first day of competition, whilst players reported progressively smaller changes in perceived well-being between matches on the second day (Fig 3A). This may be explained by peak match work-rates during match 1 (Fig 2) resulting in greater post-match increases in perceived fatigue and general muscle soreness or due to other factors, such as match outcome, that affected players’ mood and stress levels (Fig 4). Regardless, the majority of perceived fatigue reported by players following the final match can be explained by worsened levels of fatigue and general muscle soreness, which were decreased following the first match (ES = 1.27–1.36) and remained suppressed compared to pre-tournament values prior to the second day of competition (ES = 0.86–0.99). Low- and high-fitness groups reported similar changes in fatigue and general muscle soreness during the tournament, although the low-fitness group reported substantially lower scores than the high-fitness group following match 4 perhaps resulting from greater perceived match-intensity (Fig 4). Further, there were moderate to large correlations (r = 0.46 to 0.52) between changes in perceived well-being and changes in mean HR, distance/min and Playerload^TM^/min (match 1 vs. match 5) suggesting that the degree of perceptual fatigue reported by players is associated with reduced match work-rates during the latter stages of tournament competition. The assessment of players’ perceived well-being using a simple and cost-effective questionnaire may provide useful information on players’ levels of fatigue that negatively impact their physical performance during tournament competition.

The low- and high-fitness groups reported similar temporal changes in jump height suggesting Yo-Yo IR2 did not influence neuromuscular fatigue responses during the tournament (Fig 3D). Players’ jump height peaked following the first match on the second day of competition (post-match 2) and did not change substantially until following the final match (Fig 3C). The small decreases in jump height following match 5 (-4.9% to -5.8%; ES = 0.49–0.52) compared to previous matches on the same day may be indicative of increased neuromuscular fatigue; possibly resulting from residual fatigue from the previous match where players reported peak total distance and session-RPE (Table 2) in combination with reduced recovery between matches (Fig 1; 90 min vs. 120–135 min). However, this is highly speculative considering jump height following the final match did not change substantially from pre-tournament values despite large increases in perceived fatigue and general muscle soreness. This finding is in agreement with previous research in tag football [6], soccer [12] and Australian football [8], that have questioned the sensitivity of jump height as a measure of neuromuscular fatigue following competitive matches. Future research that includes force-time derived measures of jump performance may provide more conclusive evidence of changes in neuromuscular function during tournament tag football.

Although this study provided several important findings, there are a number of limitations that warrant discussion. Firstly, the results of this study were based on a relatively small group of athletes (n = 12) and only included data from a single team. Therefore, results of this study may not be generalisable to other teams due to varying playing strategies or fitness levels, and may have been impacted on by factors such as tournament scheduling. We reconciled this with the knowledge that the participating team consisted of high-level tag football players that were competing at the highest standard of tag football competition in Australia. Numbers of this size are common in research using elite athlete populations and it would have been logistically difficult to include players from other teams due to equipment availability (e.g. microtechnology devices) and match scheduling. Further, during the current study data was analysed using magnitude-based inferences to account for low statistical power and best examine the practical signficance of the data [31, 32]. Another limitation of this study was the absense of force-time derived measures of vertical jump performance during the tournament. The collection of force-time derived measures, such as flight time-to-contraction time (FT: CT), may have provided a more sensitive measure of neuromuscular function following matches than jump height [8]. Future studies aiming to identify changes in neuromuscular function during tournament team sport competition are recommended to supplement jump height with force-time derived measures.

## Conclusion

This study showed tag football players are placed under considerable physical, physiological and psychological demands during tournament competition. Coaching staff can use the findings of this study to guide training loads and fatigue-monitoring practices for tag football players preparing for tournament competition. There were large decreases in perceived well-being during the tournament which was mostly attributed to increased feelings of fatigue and general muscle soreness. Further, there were decreases in perceived well-being following one match, and feelings of fatigue and general muscle soreness remained suppressed compared to pre-tournament values prior to the second day of competition. Coaching staff may wish to replicate the intensive match loads of tournament tag football with the aim of inducing training adaptations that minimise levels of perceptual fatigue during tournament competition. Importantly, the results of this study suggest players with higher Yo-Yo IR2 perform higher tournament workloads whilst experiencing similar levels of fatigue than players with lower Yo-Yo IR2. Further, Yo-Yo IR2 appears to be associated with lower perceived match-intensities and the ability to maintain match work-rates during longer field rotations during tag football match-play. It is recommended that coaching staff prescribe training focused on improving players’ Yo-Yo IR2 to maximise physical performance and promote similar, or indeed reduced fatigue-levels during tournament tag football.
